# Focusing on Patient Needs and Preferences May Improve Genetic Counseling for Colorectal Cancer

**DOI:** 10.1007/s10897-012-9519-5

**Published:** 2013-02-01

**Authors:** Simone Salemink, Nicky Dekker, Carolien M. Kets, Erica van der Looij, Wendy A. G. van Zelst-Stams, Nicoline Hoogerbrugge

**Affiliations:** 1 Department of Human Genetics 836, Radboud University Nijmegen Medical Centre, P.O. Box 9101, 6500 HB Nijmegen, The Netherlands; 2 Scientific Institute for Quality of Healthcare, Radboud University Nijmegen Medical Centre, Nijmegen, The Netherlands; 3 Department of Medical Oncology, Radboud University Nijmegen Medical Centre, Nijmegen, The Netherlands

**Keywords:** Genetic counseling, Patient preferences, Colorectal neoplasms

## Abstract

During cancer genetic counseling, different items which counselors consider important are discussed. However, relatively little empirical evidence exists regarding the needs and preferences of counselees. In this study needs and preferences were assessed from counselees with a personal and/or family history of colorectal cancer (CRC), who were referred for genetic counseling regarding CRC. They received a slightly modified version of the QUOTE-GENE^ca^ questionnaire prior to their first visit to the Hereditary Cancer Clinic. Response rate was 60 % (48/80 participants). Counselees rated the importance of 45 items assessing their needs and preferences regarding the content and process of genetic counseling. Participants rated the items regarding discussion of information about their familial CRC risk (100 %) and preventive options (98 %) as important or very important. Fewer participants rated items concerning general information on genetics as important. Sensitive communication during counseling was considered very important by a large percentage of counselees. Generally, no major differences were seen between participants in relation to individual characteristics. Our data suggest that focusing on familial CRC risk and surveillance options, in combination with sensitive communication may lead to better satisfaction with genetic counseling.

## Introduction

The lifetime risk of developing colorectal cancer (CRC) in Western society is approximately 5–6 % (Jemal et al. 2009; The Netherlands Cancer Registry). The majority of these patients have sporadic CRC, while familial and hereditary cancers account for approximately 15 % to 20 % of all CRCs (de Jong and Vasen 2006; Grover et al. 2004; Lynch and de la Chapelle 2003). In these families, healthy relatives of CRC patients have an increased risk of developing CRC themselves, which may be prevented by surveillance colonoscopies (Dove-Edwin et al. 2005; Jarvinen et al. 2000).

Familial CRC risk is generally divided into three groups, based on cumulative lifetime risks of developing CRC (Dutch Society for Clinical Genetics 2008):

- Average—familial CRC risk below 10 %
- Moderate—familial CRC risk of 10–15 %
- High—familial CRC risk above 15 %

For patients with a high familial CRC risk, referral for genetic counseling is recommended. During genetic counseling, patients and their relatives receive information on the consequences and nature of hereditary CRC, the most common being Lynch syndrome. In a subset of families, genetic analysis for Lynch syndrome is performed by microsatellite instability (MSI) and/or DNA-testing. Based on these test results and the interpreted family history, a tailored surveillance plan is proposed by the clinical geneticist or genetic counselor. Regular surveillance by colonoscopy is crucial for individuals with an increased familial CRC risk, as it can reduce CRC-related morbidity and mortality up to 80 % (Dove-Edwin et al. 2005; Jarvinen et al. 2000).

Currently, most at-risk individuals for CRC have not had a surveillance colonoscopy (Longacre et al. 2006). Amongst other reasons, one underlying cause seems to be the inadequate recognition of individuals at risk for CRC. To improve this aspect of healthcare, a new Dutch guideline on familial and hereditary CRC was introduced in 2008, in which clinicians have new tasks in calculating, interpreting, and communicating familial CRC risk (Dutch Society for Clinical Genetics 2008). These tasks should lead to better recognition of individuals at an increased familial CRC risk, enabling them to undergo surveillance colonoscopies.

Family history, the basis of clinical indication, does not predict by itself whether an individual undergoes CRC surveillance or not (Griffith et al. 2008). Therefore, there must be other barriers for at-risk individuals to undergo surveillance. Some of these barriers might even be related to socio-demographic factors, such as age (Bleiker et al. 2005) or education level (Halbert et al. 2004). However, several studies showed that recommendation for CRC screening by a healthcare provider is a significant predictor of timely screening for individuals at increased risk for CRC (Griffith et al. 2008; Lewis et al. 2006). Interventions also seem to be most successful when recommendations are tailored to individuals or communities (Powe et al. 2010). From this perspective, tailoring genetic counseling to the needs and preferences of the counselee may lead to better understanding of the importance of CRC screening and thus increase surveillance uptake.

The traditional model for cancer genetic counseling focuses on the systematic discussion of cancer genetics, medical facts, risks of developing cancer, psychosocial consequences, and surveillance policy. This model is based on general key goals of genetic counseling regarding hereditary cancer as previously established by the counselors: (a) identifying individual needs and concerns of the counselee, (b) providing information on genes and chromosomes, (c) giving an individual risk assessment in the context of supportive interaction, and (d) discussing the pros and cons of genetic testing and drawing up a surveillance plan (Lobb et al. 2001). During counseling, careful attention must also be given to the patient’s autonomy and ability to make well-informed decisions regarding testing and adoption of preventive strategies (Robson et al. 2010; Lobb et al. 2002).

Counselors’ main goals of genetic counseling for hereditary CRC are well known; however, to our knowledge, the needs and preferences of counselees have scarcely been assessed. Previous studies investigated women’s preferences for the genetic counseling aspects of providing cancer, gene, and risk information (information); giving advice about cancer surveillance (surveillance); preparing for genetic testing (preparation); and assistance with decision-making (direction). The researchers found that women who were offered *BRCA1/2* testing had the highest preference for getting information and lowest preference for direction (Apicella et al. 2005; Peacock et al. 2006). Counselees may also need information about the genetic counseling procedure prior to a first counseling visit (Pieterse et al. 2005b).

Yet, in order to provide optimal tailoring of genetic counseling for hereditary CRC, further investigation of specific needs and preferences of the counselee and their relation to socio-demographic factors is required. Knowing what patients need and prefer enables the counselor to tailor information on a surveillance plan as much as possible. Such a personalized recommendation for CRC screening by a healthcare provider may increase the probability that those individuals at risk for CRC will undergo timely surveillance.

This exploratory pilot study was performed to answer the following research questions: 1) During genetic counseling, which counseling items and communication strategies are important to counselees suspected of hereditary CRC? and 2) Are counselees’ preferences related to their individual characteristics such as medical history, genetic counseling history, gender, and age?

## Methods

### Participants

Counselees referred to the Hereditary Cancer Clinic of the Radboud University Nijmegen Medical Centre, the Netherlands, between May 2010 and July 2010 for genetic counseling on hereditary colorectal cancer (CRC) were eligible for this study. Inclusion criteria were (a) referral for genetic counseling for familial CRC or Lynch syndrome, and (b) age 18 years and above. Counselors included both clinical geneticists and genetic counselors. Due to ethical reasons, no data were collected from non-participants.

### Procedures

To evaluate counselees’ requirements concerning genetic counseling in case of hereditary cancer, an instrument called QUOTE-gene^ca^ was developed by Pieterse et al. (Pieterse et al. 2005a). In the current study, counselees received a standard invitation to the Hereditary Cancer Clinic, as well as a pre-visit anonymous questionnaire based on QUOTE-gene^ca^. Counselees were asked to return the questionnaire within 1 week. Clinical records were consulted to determine the medical status of the participant (affected or unaffected with CRC) and whether the counselee was the first in the family seeking genetic advice (index patient) or sought presymptomatic testing (in case of a known mutation in the family). Mutation carriers were offered surveillance by regular colonoscopies. Non-carriers (true negatives, being patients in whom a known family mutation is not found) were told that undergoing surveillance was unnecessary. If there was no known family mutation and no mutation was found in the patient, either no surveillance plan or a less intensive surveillance plan was drawn up according to national guidelines for familial CRC, based on family history.

### Questionnaire

The first part of the pre-visit questionnaire assessed gender, age, education level and nationality. The main part of the questionnaire was based on the QUOTE-gene^ca^ questionnaire (Pieterse et al. 2005a), which in turn is derived from the QUOTE scale (‘Quality of Care Through the Patients’ Eyes’) (Sixma et al. 2000). The QUOTE-gene^ca^ questionnaire intends to measure needs and preferences in genetic counseling for hereditary cancer. This questionnaire has been shown to capture relevant issues of concern with a high internal consistency, and is associated with previously validated measures of coping style and distress (Pieterse et al. 2005a). Some items were rephrased, without changing the content of each item. For example, the item “my risk or the risk for my family” was split into two separate items: “my own risk” and “the risk for my family.” Also, an item about “the option of additional support by a social worker” was added. As these are minor changes, we expect the psychometric properties of our questionnaire to be comparable to the original QUOTE-gene^ca^ questionnaire.

The questionnaire contained a list of cancer-specific items and a list of generic items. Cancer-specific items included “own risk of developing cancer,” “determination and meaning of being a carrier of a cancer gene,” “emotional aspects for counselee and family,” and “heredity of cancer in general.” Generic items included “procedural aspects of counseling,” “sensitive communication,” “emotional support,” and “assessment of susceptibility to the disease.” Counselees rated the importance of each item using a four-point, Likert-type scale (1 = not important, 2 = fairly important, 3 = important, 4 = extremely important). For data analysis, ratings 1 and 2 were merged to “(not or fairly) important” and ratings 3 and 4 to “(very) important.”

### Data Analysis

The interrelations between patients’ needs and preferences and their individual characteristics were compared using cross tabs and the Fisher’s Exact test. Individual characteristics used in the statistical analyses were: gender (men *versus* women), age (<35 years *versus* aged 35–50 years, *versus* >50 years), medical status (affected with CRC *versus* unaffected with CRC), education (low *versus* middle *versus* high) and medical background (medical background *versus* no medical background). Education levels were subdivided into “low” (primary school), “middle” (junior and senior secondary vocational education), and “high” (higher vocational education and university education). All computations were done with the SPSS statistical package (release 16.0). Two-sided *p*-values below 0.05 were considered to be statistically significant.

## Results

### Participants

Forty-eight of 80 counselees (60 %) participated in this study. Table 1 shows the baseline characteristics of these 48 participants. There were 22 men (46 %) and 26 women (54 %). Their mean age upon completion of the questionnaire was 51.6 years (SD = 11.3; Range: 19–72).

**Table 1:** Participant baseline characteristics (*n* = 48)

	*n* ^a^	%
Age (years)		
Mean (s.d.)	51.6 (11.3)	
Range	19–72	
Gender		
Men	22	46
Women	26	54
Kind of referral		
First in family seeking advice (index)	40	83
Presymptomatic	7	15
Personal medical history		
Participant affected with CRC	15	31
Participant unaffected with CRC	33	69
Education^b^		
Low	15	31
Middle	20	42
High	12	25
Social status		
Living together (cohabitation, married)	39	81
Living alone (single, widow, divorced)	9	19
Medical background		
No	40	83
Yes	7	15
Nationality		
Dutch	46	96
Other	2	4

^a^Sample sizes vary due to missing data; ^b^Low = primary school; Middle = junior and senior secondary vocational education; High = higher vocational education and university education

### Needs and Preferences Prior to the First Visit

As shown in Table 2, counselor provision of information about the counselee’s own risk of developing (a second) CRC, as well as this risk for relatives, was rated as important by every participant (100 %). Almost every participant wanted to know what to do if they had an increased risk of developing CRC (98 %). Counselor explanation of emotional consequences for themselves and for their family were rated as (very) important by 70 % and 81 % of participants, respectively. Fewer participants considered the items about heredity of cancer in general as (very) important, e.g. the prevalence of cancer in the Netherlands (35 %).

**Table 2:** Frequencies of counselees’ ratings of cancer-specific needs and preferences as important or very important (*n* = 48)^a^

During counseling, the counselor should explain…	(Very) important	
	*n*	%
How risks for myself and my family are computed	46	96
Own risk of developing cancer		
My risk of developing cancer (again)	48	100
What to do if I have an increased risk of cancer	46	98
What to do if I do not have an increased risk of cancer	36	77
Determination and meaning of being a carrier of a cancer gene		
Whether the cancer in my family is hereditary	45	94
Why I am/am not considered for further examination	44	94
What it means to be a carrier of a certain gene	43	90
Possibilities of DNA-testing	43	90
What it means to be a carrier of a cancer gene	43	90
Limitations of DNA-testing	41	85
The procedure of DNA-testing	39	81
Emotional aspects for counselee and family		
My family members’ risk of developing cancer (again)	47	100
What it means not to be a carrier of a cancer gene	40	83
Emotional consequences for my family as a result of genetic counseling	38	81
The procedure of studying the family history	35	74
Emotional consequences for myself as a result of genetic counseling	33	70
Heredity of cancer in general		
How cancer is inherited in a family	41	85
How often cancer is hereditary	34	71
Background information (chromosomes, DNA, genes)	34	71
The prevalence of cancer in the Netherlands	17	35

^a^Sample sizes vary due to missing data

With regard to generic items of genetic counseling, all participants considered it (very) important that the counselor takes them seriously, listens carefully, gives enough time and attention, involves them in decisions that are made, and provides clear and understandable explanations (100 %). These items represent sensitive communication during genetic counseling; every item in this category was rated as (very) important by all or almost every participant. Fewer participants considered discussion by the counselor of the option of additional support by a social worker (44 %), communication with family members (60 %), and talking about the emotional aspects of the diagnostic procedure (65 %) as (very) important. These items represent emotional support during genetic counseling (see Table 3).

**Table 3:** Frequencies of counselees’ ratings of generic needs and preferences as important or very important (*n* = 48)^a^

During counseling, the counselor should…	(Very) important	
	*n*	%
Provide me with clear and understandable explanations	48	100
Sensitive communication		
Take me seriously	48	100
Listen carefully	48	100
Give me enough time and attention	48	100
Involve me in the decisions that are made	48	100
Be skilled	47	98
Give advice	47	98
Give me the opportunity to ask questions	47	98
Be open to my wishes, values and my opinions	43	90
Procedural aspects of counseling		
Give medical information	46	96
Explain the procedure of genetic counseling	46	96
Be punctual with appointments	45	94
Inform me sufficiently about what to expect	44	92
Cooperate well with my other doctors, e.g. GP or specialist	44	92
Give the opportunity to ask questions at any time	44	92
Explain the roles of the providers	42	88
Tell me how much time the diagnostic procedure takes	32	67
Assessment of susceptibility to disease		
Tell me what the risk for my family is	46	96
Tell me what my risk is	44	94
Carry out a DNA-test on me or a family member	39	81
Analyze the family history	38	81
Emotional support		
Provide me (also) with written information	43	90
Reassure me	36	77
Show understanding and sympathy	36	75
Talk about the emotional aspects of the diagnostic procedure	31	65
Discuss communication with family members	28	60
Discuss the option of additional support by a social worker	21	44

^a^Sample sizes vary due to missing data

### Items Involving Cancer-Specific Topics

Unaffected participants were more likely to consider receiving information about the procedure of studying the family history (very) important compared to participants affected with cancer, *p* = 0.025. In other words, a greater percentage of healthy participants wanted to know how a genetic risk assessment is made compared to affected patients (85 % vs. 50 %, respectively). As reported previously, receiving an explanation about their own risk of developing cancer or developing cancer again was rated as (very) important by all participants. Thus, no major differences due to participants’ background variables were seen for this item. No other significant relationships were obtained for participant characteristics and their ratings of the cancer-specific questionnaire items.

### Differences on Generic Items Due to Participants’ Individual Characteristics

Sensitive communication was (very) important for almost all participants and was not significantly related to their individual characteristics. However, women more often than men rated the item ‘the counselor is open to their wishes, values and opinions’ as (very) important, *p* = 0.015 (100 % vs. 77 %, respectively). No other significant differences on general items were seen as a function of participants’ individual characteristics.

## Discussion

### Major Findings

The results of this study show that the most prevalent information topics perceived as important by counselees prior to their first genetic counseling session are their familial colorectal cancer risk and surveillance options. Additionally, sensitive communication during genetic counseling is considered of extreme importance by counselees. Fewer counselees considered emotional aspects and general information on genetics as important. Ratings of the importance of needs and preferences generally did not differ significantly between the different socio-demographic groups.

### The Meaning and Importance of These Findings

Sensitive communication during counseling was considered very important as well by a large percentage of counselees. Especially, counselees consider it very important that the counselor takes him or her seriously, listens carefully, gives enough time and attention, and involves him or her in the decisions that are made. Evidently, the interaction between counselor and counselee is viewed as fundamental for successful counseling. This was also seen in a study by Veach et al. (2007), who published the results of a consensus meeting among genetic counselors and other healthcare professionals, in which they describe a reciprocal-engagement model of genetic counseling practice including goals and strategies that can be used during the genetic counseling process.

Our results can be used to adapt future genetic counseling on hereditary CRC to the needs and preferences of the counselee. By better adapting the genetic counseling to the needs and preferences of the counselee, consultation time can be saved and used to explain important and complex issues instead, such as familial CRC risk and surveillance options for those who are at risk for CRC. The focus of genetic counseling must be on topics considered important and less on topics not considered essential by both counselor and counselee. By adapting genetic counseling to counselees’ preferences, counseling may become more effective and more attention can be given to surveillance options for those who warrant surveillance.

Although generally no significant differences in needs and preferences were found in relation to counselees’ socio-demographic characteristics, it is important to keep in mind that every counselee has specific needs and preferences based on other factors. Besides, this study showed that sensitive communication was considered very important by every counselee. Counselors should take into account what might explain observed trends in different needs and preferences. For example, presymptomatic counselees may consider emotional aspects during genetic counseling more important than patients who are the first members of a family seeking advice on hereditary CRC. These so-called index patients may not always foresee possible outcomes. In addition, in this study, healthy participants were more likely to consider it important to understand the process of genetic risk assessment compared to affected participants. It is possible that participants with CRC consider risk assessment less important because they were affected already with CRC. Also, the present findings suggest women consider it more important than men that the counselor is open to their wishes, values, and opinions. One explanation could be that men have more interest in facts and medical information, while women consider communication itself more important. In consideration of these interpersonal differences, counselors always need to verify a counselee’s personal background and adapt their counseling to their individual situation.

### Relation of the Findings to Those of Similar Studies

The results of our study are partly in line with findings from other research. Peacock et al. (2006) showed that women’s preferences include information and surveillance advice. Our study distinguishes between medical information (see Table 3) and background information (see Table 2) and shows that especially medical information is considered (very) important by 96 % of the counselees. The need for background information on chromosomes, DNA, and genes is considered (very) important by 71 % of the counselees.

Our results also concur with previous findings by Pieterse et al. (Pieterse et al. 2005a). In their study, 200 new counselees, primarily counseled for breast and colon cancer, completed a QUOTE-gene^ca^ questionnaire prior to their first consultation. They found that the patients’ preferences when interacting with the counselor were receiving information, risk and preventive strategies for oneself and/or family members and information about the procedure of genetic counseling. Less patients considered emotional support and discussing emotional aspects as very important beforehand. However, 91 % of the counselees in that study were women. This leads to difficulties with extrapolation of these findings to genetic counseling for late-onset diseases that men and women seek more equally, such as CRC. Although the power of our study was limited, no major significant differences in needs and preferences between men and women were found. This indicates that this instrument can be meaningfully adapted to counselees for other types of late-onset hereditary cancers.

In another study by Pieterse et al., 130 counselees, referred mainly for breast or colon cancer, completed a questionnaire containing the QUOTE-gene^ca^ before their first appointment at a genetic clinic (Pieterse et al. 2005b). They showed that counselees had a stronger psychosocial focus than counselors, as counselees initiated the discussion of emotional consequences of DNA testing more often than their counselor, compared to other topics assessed. However, our study shows that counselees seeking presymptomatic testing consider emotional aspects of genetic counseling more important than those who are the first in the family seeking genetic advice. Providing more information on the counseling content and procedure prior to their visit may prepare counselees for possible unforeseen consequences of genetic counseling. Furthermore, new counselees may be advised to prepare for the visit more thoroughly, allowing them to verbalize questions more frequently during consultation (Albada et al. 2011; Pieterse et al. 2005b).

### Study Limitations

The results of our study are based on a limited number of counselees from one country. In addition, 40 % of the eligible participants did not enroll in this study, possibly limiting generalizability of the results. Since no data were collected for the non-participants, the possibility of an enrollment bias cannot be excluded. However, a strength of our study is that the group of participating counselees was very diverse, for instance in age, being affected with cancer or not, education, and gender. Another limitation to consider is that, by asking preselected questions, participants get an idea of possible items being addressed during genetic counseling. The fact that these items are proposed in the questionnaire, implies that they are at least important to someone else. This may be the reason that some participants were inclined to score all topics as (very) important, which may cause a social desirability bias. Also, a four point scale without a mid-point appears to push more respondents towards the positive end of the scale (Worcester and Burns 1975). In addition, educational level may have influenced counselees’ understanding of the questionnaire items. However, the percentage of low educated participants is normal compared to the general population and almost all questions were answered in which no differences were seen among counselees with different education levels, suggesting that this was not the case.

### Practice Implications and Research Recommendations

The results of our study may contribute to optimal adaptation of genetic counseling for hereditary colorectal cancer to counselees’ needs. Focusing on familial CRC risk and surveillance options may lead to better satisfaction with genetic counseling and improved adherence to surveillance policies. However, it remains important for all counselors to keep in mind that every counselee has their own specific needs and preferences. Since there is great variability among counselees, using a questionnaire such as the QUOTE-gene^ca^ prior to the first genetic counseling session may help genetic counselors to determine which items to discuss with the counselee. However, it is necessary to explore the best content and format as well as assess the added value of this strategy. Additionally, larger studies are needed to determine whether indeed, very few differences in needs and preferences are present between counselees with different socio-demographics. It will also be necessary to explore whether patient satisfaction and surveillance uptake for colorectal cancer can indeed be improved in this manner.
